# Supplementary material for: Digital Interventions for Psychological Comorbidities in Chronic Diseases—A Systematic Review
Source: J Pers Med. 2021 Jan 6;11(1):30. doi: 10.3390/jpm11010030 (PMC7825345; doi:10.3390/jpm11010030)
Supplement: Supplementary file 1 [file jpm-11-00030-s001.zip › FigureS1_Funnel plots.pptx]

## Slide 1
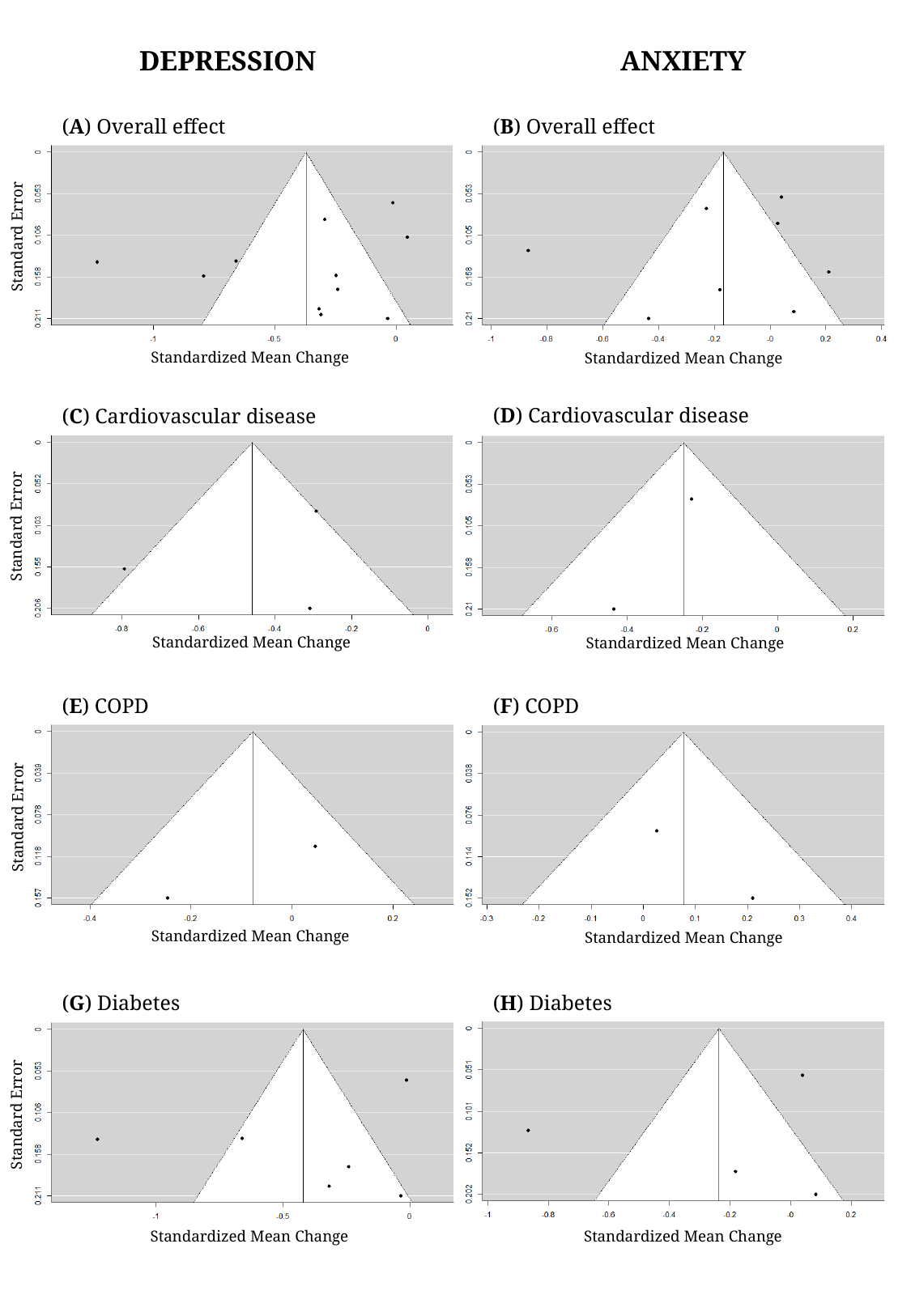

Anxiety
Depression
(A) Overall effect
(B) Overall effect
Standard Error
Standardized Mean Change
Standardized Mean Change
(D) Cardiovascular disease
(C) Cardiovascular disease
Standard Error
Standardized Mean Change
Standardized Mean Change
(E) COPD
(F) COPD
Standard Error
Standardized Mean Change
Standardized Mean Change
(H) Diabetes
(G) Diabetes
Standard Error
Standardized Mean Change
Standardized Mean Change
